# Anticancer activity of a monobenzyltin complex C1 against MDA-MB-231 cells through induction of Apoptosis and inhibition of breast cancer stem cells

**DOI:** 10.1038/srep38992

**Published:** 2016-12-15

**Authors:** Somayeh Fani, Behnam Kamalidehghan, Kong Mun Lo, Siamak Ebrahimi Nigjeh, Yeap Swee Keong, Firouzeh Dehghan, Rahman Soori, Mahmood Ameen Abdulla, Kit May Chow, Hapipah Mohd Ali, Fatemeh Hajiaghaalipour, Elham Rouhollahi, Najihah Mohd Hashim

**Affiliations:** 1 Department of Pharmacy, Faculty of Medicine, University of Malaya, 50603 Kuala Lumpur, Malaysia; 2 Medical Genetics Department, National Institute of Genetic Engineering and Biotechnology (NIGEB), Tehran-Karaj Highway, Tehran, Iran; 3 Department of Chemistry, Faculty of Science, University of Malaya, 50603 Kuala Lumpur, Malaysia; 4 Institute of Bioscience, University Putra Malaysia, 43400 Serdang, Malaysia; 5 Department of Exercise Physiology, Faculty of Physical Education and Sport Sciences, University of Tehran, 14174 Tehran, Iran; 6 Department of exercise science, Sports Center, University of Malaya, Kuala Lumpur 50603, Malaysia; 7 Department of Biomedical Science, Faculty of Medicine, University of Malaya, 50603 Kuala Lumpur, Malaysia; 8 Center for Natural Products and Drug Discovery (CENAR), Department of Chemistry, Faculty of Science, University of Malaya, Kuala Lumpur, Malaysia

## Abstract

In the present study, we examined the cytotoxic effects of Schiff base complex, [*N*-(3,5-dichloro-2-oxidobenzylidene)-4-chlorobenzyhydrazidato](o-methylbenzyl)aquatin(IV) chloride, and C1 on MDA-MB-231 cells and derived breast cancer stem cells from MDA-MB-231 cells. The acute toxicity experiment with compound C1 revealed no cytotoxic effects on rats. Fluorescent microscopic studies using Acridine Orange/Propidium Iodide (AO/PI) staining and flow cytometric analysis using an Annexin V probe confirmed the occurrence of apoptosis in C1-treated MDA-MB-231 cells. Compound C1 triggered intracellular reactive oxygen species (ROS) production and lactate dehydrogenase (LDH) releases in treated MDA-MB-231 cells. The Cellomics High Content Screening (HCS) analysis showed the induction of intrinsic pathways in treated MDA-MB-231 cells, and a luminescence assay revealed significant increases in caspase 9 and 3/7 activity. Furthermore, flow cytometric analysis showed that compound C1 induced G0/G1 arrest in treated MDA-MB-231 cells. Real time PCR and western blot analysis revealed the upregulation of the Bax protein and the downregulation of the Bcl-2 and HSP70 proteins. Additionally, this study revealed the suppressive effect of compound C1 against breast CSCs and its ability to inhibit the Wnt/β-catenin signaling pathways. Our results demonstrate the chemotherapeutic properties of compound C1 against breast cancer cells and derived breast cancer stem cells, suggesting that the anticancer capabilities of this compound should be clinically assessed.

The appearance of *cisplatin*-sensitive and *cisplatin*-resistant human tumor cell lines, and serious side effects, such as ototoxicity and peripheral neuropathy, have confirmed the remarkable clinical efficacy of cisplatin^1^,^2^. It is important to find and develop other metal-based antitumor agents that are capable of minimizing the toxic side effects of treatment. Organotin (IV) complexes with Schiff bases are non-platinum, metal-based agents that could have potential antitumor activity^3^. Organotin complexes have shown strong cytotoxicity against many human cancer cell lines^4^,^5^,^6^,^7^. The application of the organotin complexes has increased due to the variety of organic moieties and donor ligands that bind to the tin center in the structure of the complexes.

An increasing body of evidence has supported the idea that changes in the signal transduction pathways of molecular mechanisms, such as proliferation, invasion, angiogenesis, and apoptosis, are associated with the initiation of breast tumors^8^,^9^. Apoptosis is a finely tuned and well-examined mechanism that manages normal tissue development and homeostasis using a complicated genetically encoded network of molecules that participate in cell death^10^,^11^. Extreme quantities of ROS can induce damage to lipids, and proteins, leading to DNA oxidative damage and resulting in cell death^12^. Damaged DNA can prompt the activity of Bax proteins, a family of pro-apoptotic Bcl2 members, causing penetrability of the mitochondrial membrane and subsequent mitochondrial cytochrome *c* release. Cytochrome *c* triggers the activity of caspase 9, and activated caspase 9 then stimulates the activity of caspase 3. This event subsequently gives rise to the activation of the other members of the caspase cascade, which ultimately resulting in apoptotic features, such as DNA condensation and fragmentation, and membrane blabbing^13^,^14^. The Bcl2 family of proteins is one of the most important hallmarks of apoptosis, and it consists of both pro-apoptotic and anti-apoptotic proteins. Pro-apoptotic proteins, such as Bax, stimulate mitochondrial outer membrane potential, while, anti-apoptotic proteins, such as Bcl2, hinder MOMP stimulation. Thus, in the mitochondria, the fraction of Bcl2/Bax proteins is a main cause of the initiation of apoptosis by anticancer drugs^15^,^16^.

There is growing evidence that cancer stem cells (CSCs), a distinct subpopulation of tumor cells, are the predecessors and organizers of many types of cancer^17^,^18^. This idea was first established in human myeloid leukemias. Later, it was established by examining solid tumors, such as brain and breast cancers^19^. Sequential self-renewal and the differentiation of cancer stem cells explain tumor recurrence after treatment of tumors with radiation or chemotherapy, as well as the failure of current therapies to eliminate CSCs^20^. Numerous signaling pathways, such as Wnt/β-catenin, hedgehog, and Notch, control the renewal and differentiation of CSCs^21^,^22^. Bioactive dietary complexes, such as quercetin and curcumin, have the ability to target the self-renewal pathways of CSCs^23^,^24^. Continuing research into the effects of synthetic compounds against CSCs could confirm the CSC hypothesis as an effective strategy for reducing tumor resistance and relapse.

The Wnt/β-catenin signaling pathways constitute a central part of the self-renewal of breast CSCs^25^. In mammals, the activity of Wnt target genes is regulated by a combination of β-catenin and T-cell factor/lymphoid enhancer factors after the translocation of cytoplasmic β-catenin into the nucleus^21^,^26^,^27^. Intracellular β-catenin levels are modulated through the interaction of β-catenin with a complex of axin, casein kinase 1 (CKI) **a**, and adenomatous polyposis coli (APC). This interaction activates GSK3β, which results in the ubiquitin proteasome phosphorylation of β-catenin on three specific amino acids, namely Ser33, Ser3, and Thr41, and the degradation of β-catenin^21^,^26^. Glycogen synthase kinase-3 ß (GSK-3ß) is a multi-functional serine/threonine kinase. GSK-3ß was first identified as an important regulator of glycogen metabolism and the insulin signaling pathway. GSK-3ß targets more than 40 molecules, including cyclin D1 protein. The activity of GSK-3ß is inhibited by its phosphorylation at serine 9. GSK-3ß is an important manager of cell survival by negatively regulating the Wnt/ß-catenin pathway. Therefore, targeting of GSK-3ß has gained great attention in cancer drug discovery.

In this study, the *in vitro* efficacy of the organotin complex C1 against MDA-MB-231 breast CSCs and its potential to suppress the Wnt/β-catenin signaling pathway were examined. In addition, the acute toxicity of compound C1 was assessed.

## Results

### Safety of compound C1

**Figure 1:**
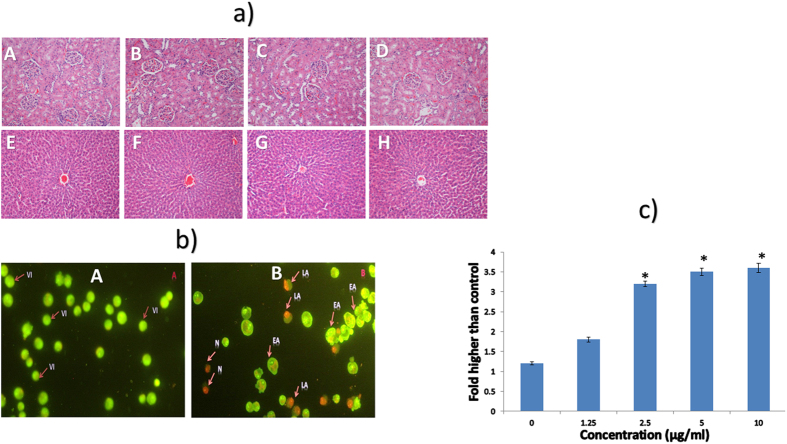
(**a**) Histological sections of liver and kidney. Histology (hematoxylin and eosin stain, 20×) of the liver (A–D) and kidney (E–H) did not show any abnormality after treatment with (B and F) 25 mg/kg, 50 mg/kg (C and G), and 100 mg/kg (**D** and **H**) of compound C1 compared to the vehicle distilled water (A and E). (**b**) AO/PI staining of untreated and treated MDA-MB-231 cells with the IC_50_ concentration of compound C1 (2.5 μg/mL) after 48 hours: (A) Untreated cells, which display VI: viable cells; (B) treated cells, which display EA: early apoptotic cells, LA: late apoptotic cells, N: necrotic cells. (**c**) Lactate dehydrogenase (LDH) assay: Significant release of LDH in the cell culture medium after treatment of MDA-MB-231 cells with different concentrations of benzyltin complex C1 for 48 hours.

The ability of a compound to cause undesirable effects after a short period of exposure defines the acute toxicity of a compound. The acute toxicity investigation of the monoorganotin Schiff base complex C1 confirmed the safety of this complex, because all of the rats survived and did not show any signs of toxicity, mortality, or behavior changes over the 14 days of the experimental period, even at high doses of 100 mg/kg. Furthermore, there were no signs of renal or hepatic toxicity in the treated animals after histological, hematological, and serum biochemical analyses were conducted (Figure 1I Tables 1, 2, 3).

### Cisplatin inhibited the growth of MDA-MB-231 cells

The IC_50_ value of compound C1 in MDA-MB-231 cells was reported as 2.5 μg/mL in a previous study^28^. The results of this study indicated that cisplatin inhibited the growth of MDA-MB-231 cells *in vitro*. The growth inhibiting effect of cisplatin on MDA-MB-231 cells was determined using an MTT assay. As shown in Table 4, cisplatin inhibited the growth of MDA-MB-231 cells with IC_50_ values of 1.3 ± 0.55 and 0.9 ± 0.31 μg/mL at 24 and 48 hours, respectively.

### Acridine orange and propidium iodide double-staining showed morphological changes in complex C1-treated cells

The results revealed that complex C1 induced apoptotic morphological changes and caused necrosis in MDA-MB-231 cells. Green and orange fluorescence was released as a result of the bond between DNA and the dual intercalating nucleic acid dyes, AO/PI. Figure 1II (A) shows untreated cells, AO (+) and PI (−), with large, green, normal nuclear structures. The morphological characteristics of different stages of cell death after 48 hours of treatment are shown in Fig. 1II (B). In Fig. 1II (B), early apoptotic cells (AO+ PI−) are identified as green, condensed nuclear structures, late apoptotic cells (AO+ PI+) are shown as reddish-orange, condensed nuclear structures, and necrotic cells (AO+ PI+) are shown as red, swollen, enlarged cells with nuclei.

### Complex C1 Induced LDH Release

The leakage of the lactate dehydrogenase (LDH) enzyme from the cytosol into the surrounding culture medium is generally regarded as an indicator of apoptosis and necrosis. An LDH assay was performed to investigate the effects of benzyltin complex C1 on the LDH activity of treated MDA-MB-231 cells after 48 hours. The results revealed that there was a significant elevation of LDH in cells treated with the IC_50_ (2.5 μg/mL) and higher concentrations of compound C1 after 48 hours of treatment (*P* < 0.05) (Fig. 1III).

### Complex C1 induced ROS production

**Figure 2:**
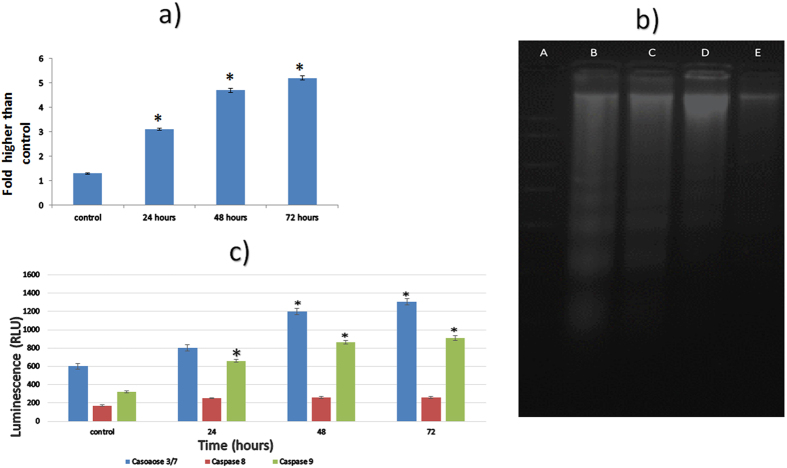
(**a**) ROS generation in treated MDA-MB-231 cells. DCF-fluorescence intensity after 2.5 μg/mL exposure of benzyltin complex C1 exposure for 24, 48, and 72 hours. (**b**) Agarose gel image of ladder formation in untreated and treated MDA-MB-231 cells with the IC_50_ concentration of compound C1 (2.5 μg/mL) after 24 and 48 hours: (A) Ladder; (B) positive control; (C) 48 hours treatment; (D) 24 hours treatmen; (E) untreated cells. (**c**) Caspases activity test: The luminescence analysis showed significant expression of caspases 3/7, 8 and 9 in compound C1-treated MDA-MB-231 cells at a concentration of 2.5 μg/mL (IC_50_) in a time-dependent manner. The data represent the means ± standard deviations (SDs) of 3 independent tests. Statistical analysis is defined as significant if *P < 0.05.

ROS are an important part of cell signaling and homeostasis; therefore, they are regularly produced and abolished in biological systems. Unnecessary increases in ROS are typically linked to potential mitochondrial membrane damage, apoptosis, and cell death. The production of intracellular ROS in C1-treated MDA-MB-231 cells was assessed using 2′, 7′dichlorofluorescein diacetate (DCFH-DA). H_2_O_2_ was used as a positive control because it is a typical ROS producer. Compared to the negative control, treatment with complex C1 induced a significant increase in ROS production in a time-dependent manner (Fig. 2I). The excessive increase in ROS production in cells treated with an IC_50_ concentration of C1 (2.5 μg/mL) might indicate the cytotoxic activity of compound C1 via the activation of mitochondria-initiated events.

### Determination of the mode of cell death by DNA laddering assay

DNA degradation in a ladder-like pattern is a characteristic of cell death caused by ROS. According to the morphological changes observed after AO/PI staining, DNA fragmentation, as a typical morphologic feature of apoptosis, was detected following the treatment of cells with compound C1 for 24 and 48 hours. As observed in Fig. 2II, the cytotoxic effects of compound C1 triggered the formation and appearance of apoptotic DNA fragments on agarose gel, which further confirmed that the C1 cytotoxic induction of MDA-MB-231 cells was mediated through the apoptosis pathway.

### Activation of caspases 3/7 and 9 induced by complex C1

The caspases are important biochemical features of apoptotic signaling. The apoptotic response of MDA-MB-231 cells to complex C1 was examined by quantifying the activity of caspases 3/7, 8, and 9. All of the caspase activities increased in the C1-treated cells in a time-dependent manner. As shown in Fig. 2C, caspase 3/7 increased dramatically after 48 and 72 hours of treatment, while caspase 9 increased significantly after 24, 48 and 72 hours of treatment (*P* < 0.05). Caspase 8 also showed an increasing trend following 24 hours of treatment; however, this increase was not statistically significant (Fig. 2III).

### Induction of PS externalization by compound C1

**Figure 3:**
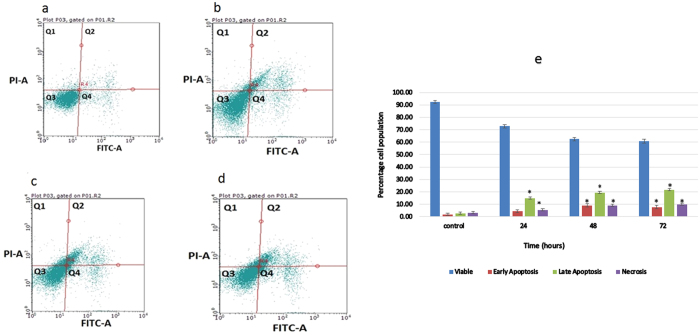
Flow cytometric analysis of Annexin V in MDA-MB-231 cells. **(a)** Untreated MDA-MB-231 cells; **(b)**, **(c)** and **(d)** show treated with compound C1 at 2.5 μg/mL for 24, 48, and 72 hours of treatment, respectively. **(e)** Representative bar chart. The data represent the means ± standard deviations (SDs) of 3 independent tests. Statistical analysis is defined as significant if *P < 0.05.

The translocation of phosphatidylserine (PS) from the interior layer of the plasma membrane to the exterior cell surface is a typical marker of early apoptosis. An Annexin-V-FITC kit was used to investigate further the apoptotic effects of complex C1 on MDA-MB-231 cells. The cells were treated with compound C1 at an IC_50_ concentration of C1 (2.5 μg/mL) for 24, 48, and 72 hours. Flow cytometry investigation revealed that apoptosis was induced in a time-dependent manner (Fig. 3a–d). For the untreated cells, the percentages of viable, early apoptotic, late apoptotic, and necrotic cells were 92.34%, 1.83%, 2.60%, and 3.23%, respectively. However, after 24, 48, and 72 hours of treatment with compound C1, the number of cells in all three different modes of cell death increased significantly. The population of early apoptotic cells (Annexin V-positive, PI-negative) increased significantly to 4.95%, 8.99%, and 7.68% after 24, 48, and 72 hours, respectively (*P* < 0.05). Fig. 3e shows that the percentage of late apoptotic cells (positive for both Annexin V and PI) increased significantly from 2.60% in untreated cells to 15.45, 19.45 and 21.63% for cells subjected to 24, 48, and 72 hours of treatment, respectively. The population of viable cells (Annexin V-negative and PI-negative) decreased to 60.79% at 72 hours after treatment, and there was significant growth in necrotic cells (Annexin V-negative, PI-positive), ranging from 3.23% for the control group to 9.90% in the cells that were treated for 72 hours (Fig. 3e).

### Complex C1 induced G0/G1 cell cycle arrest

**Figure 4:**
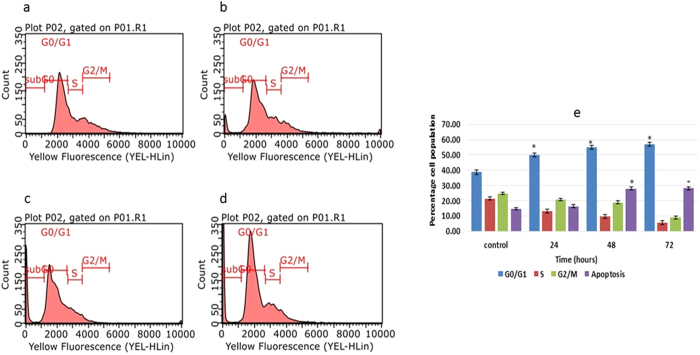
Effect of compound C1 on the cell cycle distribution of MDA-MD-231 cells. (**a**) Untreated MDA-MD-231 cells (control). After treatment with IC_50_ concentration of complex C1 (2.5 μg/mL) for (**b**) 24, (**c**) 48 and (**d**) 72 hours, flow cytometry analysis was performed on treated and untreated MDA-MD-231 cells. (**e**) The quantitative analysis indicated cell cycle arrest at the G0/G1 phase. The data represent the means ± standard deviations (SDs) of 3 independent tests. Statistical analysis is defined as significant if *P < 0.05.

PI staining and flow cytometry analysis were performed to investigate the cell cycle phase distribution in C1 MDA-MB-231 treated cells, after 24, 48 and 72 hours of treatment. According to our results displayed in Fig. 4, there was G0/G1 phase arrest in a time-dependent model from 24 to 72 hours that accounted for 49.96%, 54.81%, and 56.81% after 24, 48 and 72 hours of treatment, respectively. In addition, the sub-G0/G1 phase cell percentages also dramatically increased from 14.48% in the control cells to 28.66% and 29% after 48 and 72 hours of exposure to compound C1 (*P* < 0.05). As shown, the cell population decreased in the S phase and the G2/M phase from 21.26% to 5.53% and 24.61% to 8.97%, respectively (Fig. 4).

### Mitochondria-initiated events induced in complex C1 and cisplatin treated-cells

**Figure 5:**
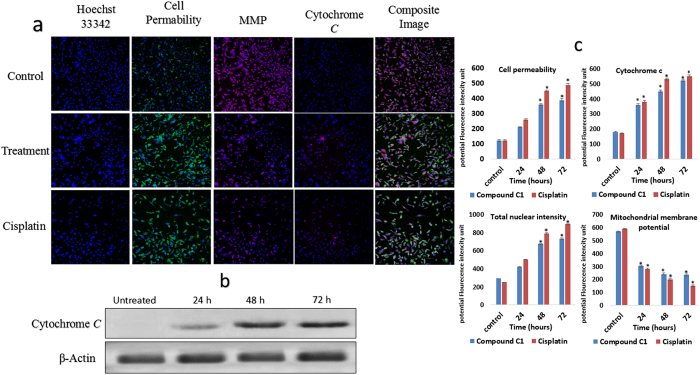
(**a**) Representative images of MDA-MB-231 cells (magnification 20×). Untreated and treated MDA-MB-231 cells with 2.5 μg/mL (IC_50_) compound C1 and 0.9 μg/mL (IC_50_) cisplatin were stained with Hoechst 33342, cell membrane permeability, cytochrome *c* and MMP dyes. Treated MDA-MB-231 cells with compound C1 and cisplatin produced a marked decrease in MMP and a noteworthy elevation in total nuclear intensity, membrane permeability and cytochrome *c.* (**b**) Western blot analysis of cytosolic cytochrome *c*: Cytochrome *c* Releasing Apoptosis Assay Kit was used to evaluate the release of cytochrome *c* from the mitochondria to cytosol in untreated and treated MDA-MB-231 cells with 2.5 μg/mL (IC_50_) compound C. The result showed notable release of cytochrome ***c***in cytosol after 24, 48, and 72 ours treatments. The data represent the means ± standard deviations (SDs) of 3 independent tests. Statistical analysis is defined as significant if *P < 0.05. (**c**) Quantitative analysis of multiparameter cytotoxicity assay in MDA-MB-231 cells: Simultaneous alterations in total nuclear intensity, cell permeability mitochondrial membrane potential, and cytochrome *c* release were quantified in MDA-MB-23 cells. After treatment with compound C1 (at IC_50_ concentration of 2.5 μg/mL), and cisplatin (at IC_50_ concentration of 0.9 μg/mL), statistically significant loss of mitochondrial membrane potential and an obvious increase in total nuclear intensity, cell permeability, and cytochrome *c* release from mitochondria were observed.

Six independent factors involved in apoptosis processes, including cell loss, the size of nuclear and morphological alterations, alterations in mitochondrial membrane potential, cytochrome *c* release, and changes in cell penetrability, were measured for the C1-treated MDA-MB-231 cells and cisplatin-treated MDA-MB-231 cells after 24, 48, and 72 hours using ArrayScan HCS system (Cellomics). The results revealed that MMP decreased significantly after 24, 48, and 72 hours of treatment, as shown by a decrease in pink fluorescence intensity. Cytochrome *c* translocation from the mitochondria to the cytosol during apoptosis increased significantly. This increase was presented as an increase in dark blue fluorescence intensity. Following treatment, significant growth in total nuclear intensity and cell permeability was clearly observed following exposure of MDA-MB-231 cells to compound C1 for 48 and 72 hours (*P < 0.05), which presented as green fluorescence (Fig. 5a). Additionally, cytosolic cytochrome *c* was measured by western blot after extraction. The result showed the release of cytochrome *c* in cytosol of C1-treated MDA-MB-231 cells after 24, 48, and 72 hours treatment, while no expression of cytochrome *c* was observed in untreated cells. (Fig. 5b). Moreover, cisplatin-treated cells at 0.9 μg/mL concentration showed the significant reduction in MMP and a remarkable increase in cytochrome c release, cell permeability, and total nuclear intensity (Fig. 5c). Comparison between results from C1-treated MDA-MB-231 cells and cisplatin-treated MDA-MB-231 cells indicate that compound C1 has almost similar effect on mitochondria-initiated events in MDA-MB-231 cells as positive control drug, cisplatin, has (Fig. 5c).

### Gene and protein expression of apoptotic markers in complex C1-treated cells

**Figure 6:**
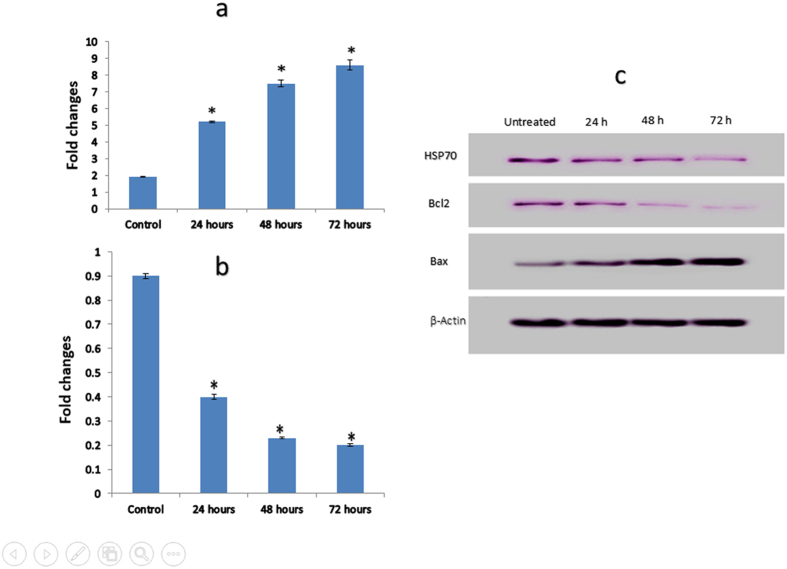
(**a and b**) Quantitative study of apoptosis markers gene expression. The expression of Bax **(a)** and Bcl2 **(b)** mRNA in MDA-MB-231 cells treated with 2.5 μg/mL (IC_50_) of compound C1 after 24, 48, and 72 hours. The results revealed a significant elevation and decrease in expression of Bax and Bcl2 genes. **(c)** Western blot analysis of apoptotic pathway hallmarks: Treatment with 2.5 μg/mL (IC_50_) of compound C1 after 24, 48, and 72 hours increased protein expression levels of Bax, and decreased Bcl2, and HSP70. The data represent the means ± standard deviations (SDs) of 3 independent tests. Statistical analysis is defined as significant if *P < 0.05.

Members of the Bcl2 family, including Bcl2 and Bax, play critical roles in the regulation of apoptosis. The mRNA expression alteration of Bax and Bcl2, pro-apoptotic, and anti-apoptotic genes was measured in treated MDA-MB-231 cells by real-time PCR analysis. The analysis revealed a significant increase in Bax expression after 24, 48, and 72 hours (*P* < 0.05), compared to the untreated cells (Fig. 6a). As shown in Fig. 6b, the expression of the anti-apoptotic protein Bcl2 significantly decreased after 24, 48, and 72 hours of treatment (*P* < 0.05). The significant changes in these proteins at the gene expression level caused us to examine their function in the C1 mechanism underlying induced apoptosis by analyzing their expression at the protein level. The expression of Bcl2, Bax and HSP70 in C1-treated MDA-MB-231 cells was tested after being standardized to β-actin. The expression of Bcl-2 and HSP70 was considerably reduced, whereas the rate of Bax protein increased significantly in a time-dependent manner (Fig. 6c). Mitochondria-mediated apoptosis is related to the release of cytochrome *c* from the mitochondria to cytosol. Based on our results, compound C1 has increased cytosolic cytochrome *c* in a time-dependent manner.

### CD markers in breast CSCs

**Figure 7:**
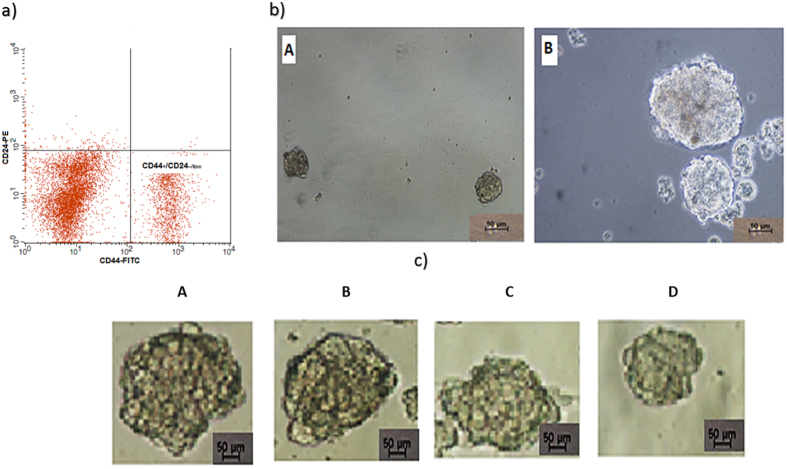
(**a**) MDA-MB-231 cancer stem cells identification. MDA-MB-231 CSCs were recognized by expression of CD44+ and low expression of CD24-/low in quadrant examination (CD44+/CD24-/low). (**b**) Mammosphere formation of MDA-MB-231 cancer stem cells: (A) and (B) show the size of mammospheres containing MDA-MB-231 cancer stem cells on day 5 and day 7, respectively. (**c**) reduction in the size of primary mammospheres in treated MDA-MB-231 cancer stem cells: Cancer stem cells were treated with complex C1 at 1 (B), 2 (C), and 4 (D) μg/mL concentrations. A indicates untreated cells. Complex C1 reduced the size of the primary mammospheres in treated MDA-MB-231 cancer stem cells. Each experiment was performed in triplicate.

As shown in Fig. 7I, quadrant analysis identified breast CSCs from MDA-MB-231 cells. These cell populations were isolated and identified based on the expression of CD44+/CD24-/low, which are cell surface markers that indicate the presence of BCSCs.

### Inhibition effect of complex C1 on mammosphere development

**Figure 8:**
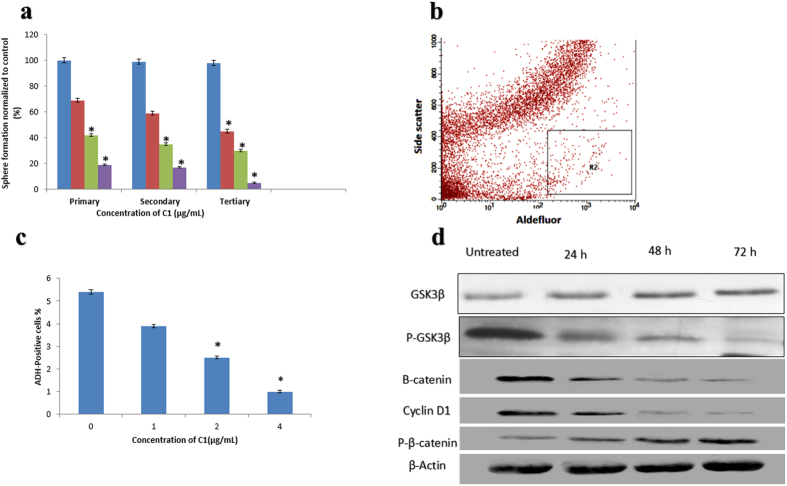
(**a**) Inhibition effect of compound C1 on primary, secondary, and tertiary mammosphere-forming units. In the absence of drug, the second and third passages derived from Complex C1-treated primary mammospheres yielded smaller numbers of spheres in comparison with the controls. The size of the mammospheres was estimated using V = (4/3) πR3. Complex C1 hinders mammosphere formation and prevents self-renewal of MDA-MB-231 primary, secondary, and tertiary mammosphere-forming units. **(b and c)** Aldefluor assay of MDA-MB-231 cancer stem cells: Single cells obtained from cell cultures were incubated for 50 minutes at 37 °C in Aldefluor assay buffer comprising an ADH substrate, BODIPY-aminoacetaldehyde (1 μmol/L per 1 × 10^6^ cells). **(b)** Cell population (R2) with high ALDH activity was informed to develop mammary stem/progenitor cells. **(c)** Quantitative analysis of the inhibitory effects of C1 on ALDH-positive cell populations MDA-MB-231. Cancer stem cells were treated with complex C1 at 1, 2, and 4 μg/mL concentrations for 4 days and were subjected to an Aldefluor assay and flow cytometry analysis. Complex C1 decreased the percentage of ALDH-positive cells. (**d**) Western blot analysis of the Wnt/β-catenin self-renewal pathway markers: Treatment with 2.5 μg/mL (IC_50_) of compound C1 after 24, 48, and 72 hours increased protein expression levels of P-β-catenin and decreased β-catenin, cyclin D1, and P-GSK3β. The expression level of GSK3β was almost unchanged. The data represent the means ± standard deviation (SDs) of 3 independent tests. Statistical analysis is defined as significant if *P < 0.05.

Formed mammospheres were initially characterized using a Nikon Eclipse TE2000-S microscope. The numbers of mammospheres were determined, and photos were attained using MetaMorph software, version 7.6.0.0. As shown in Fig. 7II (A and B), non-adherent spherical clusters of cells formed after culturing the stem/progenitor cells from MDA-MB-231 cells in serum-free medium. To study the inhibitory effects of compound C1 on mammosphere formation, MDA-MB-231 CSC spheres were exposed to different concentrations of compound C1. Our results clearly demonstrated a decrease in the size of mammospheres after treatment and a significant decrease in the number of mammosphere formed from control to higher concentrations of treatment (*P* < 0.01) (Fig. 7III A–D). Additionally, when cells from the original plating (Passage 1) were collected and observed for the subsequent formation of mammospheres (Passage 2), they could not form secondary spheres (Fig. 8a).

### Inhibition effects of compound C1 on ALDH-positive cells

To examine whether compound C1 suppressed the growth of MDA-MB-231 CSCs, the activity of ALDH enzyme was assessed by the Aldefluor assay. ALDH-positive MDA-MB-231 cell populations are known to develop breast stem/progenitor cells and to regulate the self-renewal of MDA-MB-231 CSCs. According to our results, treatment with different concentrations of compound C1 (1, 2 and 4 μg/mL) decreased the population of ALDH-positive MDA-MB-231 CSCs cells by more than 25%, 50%, and 75%, respectively (Fig. 8b and c). It is worth mentioning that compound C1 diminished the population of MDA-MB-231 CSCs at various concentrations of treatment, but it did not decrease the bulk of the population of MCF-10A cells, suggesting that compound C1 has the selective ability to target MDA-MB-231 CSCs.

### The expression of Wnt/β-catenin self-renewal pathway’s markers in complex C1-treated cells at protein level

The importance of the Wnt/β-catenin pathway in cancer has been confirmed by frequent studies over the last 5 years^29^. Many studies have demonstrated that the activation or inactivation of Wnt/β-catenin pathway regulators has a direct effect on the development of breast cancer^30^. Treatment of MDA-MB-231 cells with compound C1 reduced the expression of β-catenin, cyclin D1, and p-GSK3-β, while the expression of p-β-catenin (Ser33/Ser37/Thr41) increased. The expression level of GSK3-β in treated MDA-MB-231 cells remained almost unchanged (Fig. 8d).

## Discussion

The association of the efficacy of current cancer treatments and apoptosis has been confirmed by several molecular and histological tests^31^. Consequently, many researchers are concentrating on the discovery and development of new anticancer drugs with apoptotic-inducing properties. This study was conducted to evaluate the *in vitro* effects of complex C1, a new monoorganotin Schiff base complex, against MDA-MB-231 cells and derived MDA-MB-231 breast cancer stem cells/progenitors. One of the primary goals in the field of cancer drugs is the development of agents with more specificity for cancer cells and less toxicity to humans^32^,^33^.

According to the MTT assay published in a previous study, the IC_50_ value of compound C1 for MDA-MB-231 cells was 2.5 μg/mL. This value was greater than 30 in a normal human hepatic cell line, WRL-68. The acute toxicity experiment demonstrated the safety of this agent. Apoptosis is linked to many biochemical alterations in cells, such as DNA fragmentation, changes in mitochondrial membrane potential, changes in caspase activities, and a loss of membrane integrity. Florescence analysis showed apoptosis in treated MDA-MB-231 cells. Staining of treated cells with AO/PI, one of the most common florescent dyes, showed morphological features of necrotic cells and cells in different phases of apoptosis.

The LDH assay, which was based on the release of the lactate dehydrogenase enzyme into the culture medium, further confirmed the significant cytotoxic effects of C1 against treated MDA-MB-231 cells. The LDH release increased significantly after treatment in a concentrated manner, indicating a loss of membrane integrity and cell death^33^. Additionally, the translocation of phosphatidylserine (PS) from the internal layer of the plasma membrane to the external cell surface is a marker of early stages of apoptosis^34^,^35^.

FITC-conjugated Annexin V-PS binding can be detected by flow cytometry analysis, which indicates the translocation of PS and consequent loss of the plasma membrane^36^. Significant increases in early and late apoptotic cell populations after 24, 48 and 72 hours of treatment and necrotic cells after 48 and 72 hours of treatment were consistent with the results of the LDH assay regarding death through the loss of cell membrane symmetry. The effect of C1 on the release of cytochrome *c* from the mitochondria into the cytoplasm and the consequent caspase activity were investigated to detect C1-induced apoptosis. Additionally, the production of ROS after treatment and the contribution of pro-apoptotic and anti-apoptotic proteins in the regulation of C1 apoptosis induction were investigated at both the gene and protein levels. The crucial role of mitochondria in C1-apoptosis induction was found based on a significant reduction in MMP and a subsequent significant increase of cytochrome *c* in treated MDA-MB-231 cells. It was evident that ROS contributed to the oxidation of mitochondrial pores, loss of MMP, and cytochrome *c* relocalization^11^,^13^,^37^. Treatment with C1 significantly increased ROS production in a time-dependent manner.

Caspase activity is the main factor in the regulation of the apoptosis mechanism downstream of caspase 9 activation, which occurs as a result of cytochrome *c* release, which in turn leads to the activation of effector caspases, including caspase 3 and caspase 7^38^,^39^,^40^,^41^. Caspase 8 activation, which is a major participant in extrinsic pathways, is the result of cell death receptors, such as Fas and TNF-α. Activated caspase 8 also leads to the activity of effector caspases, including caspase 3 and caspase 7^13^,^41^,^42^,^43^. The treated MDA-MB-231 cells experienced a significant increase in caspase 9 after 24, 48, and 72 hours of treatment. The increase in caspase 3/7 was significant after 48 hours of treatment. Although there was an increase of caspase 8 in treated cells compared to untreated cells, this alteration was not significant. There was evidence of the downregulation of Bcl2, a pro-apoptotic protein, and the upregulation of Bax, a pre-apoptotic protein, which have central functions in changing mitochondrial roles and the release of cytochrome *c*^41^,^42^,^43^,^44^,^45^. Heat shock protein 70 (HSP70), a significant chaperone, is an anti-apoptotic protein that inhibits the main factors of the apoptosis pathway^46^,^47^. The protein expression of HSP70 was downregulated in a time-dependent manner in C1-treated cells. Treatment with C1 increased Bax and decreased Bcl2 significantly at both the gene and protein levels. In addition, flow cytometry study of complex C1 in cell cycle distribution showed G0/G1 arrest of the treated cells. The cell accumulation in the G0/G1 phase was increased significantly. Previous studies have found that CSCs must be eradicated to eliminate tumors and to prevent relapse^48^,^49^, suggesting that treatments that targeted cancer cells together with CSCs have provided a valuable strategy for cancer treatment^50^,^51^. Screening of breast cancer stem cells might be based on techniques in which CSCs are enriched. These methods include cell surface marker-based assays, such as CD44+/CD24-/low and ALDH, or mammosphere formation from cells, which is also an effective method to isolate and characterize BCSCs^52^,^53^. *In vitro* floating spherical colony formation is based on the ability of stem cell-like/progenitor cells to survive and the inability of differentiated cells to proliferate in serum-free conditions^54^,^55^. Cells were plated on ultra-low attachment plates and treated. Mammospheres composed from the first plating (P1) were collected and replated to study the formation of mammospheres in P2 and P3. Our findings were consistent with the results published in prior studies, which found that mammospheres were composed principally of CSCs^21^,^56^. Complex C1 significantly inhibited mammosphere development and suppressed the self-renewal of primary, secondary, and tertiary mammosphere-forming units. Using cell markers, such as CD44+/CD24-/low and ALDH positivity, is an additional method that differentiates between BCSCs and breast cancer cells. Flow cytometric studies of MDA-MB-231 cells found a subpopulation of CD44+/CD24- cells. Consistent with previous studies, an Aldefluor assay of MDA-MB-231 cells revealed the expression of the aldehyde dehydrogenase enzyme. Based on our results, complex C1 has the ability to selectively suppress ADH-positive cancer cells *in vitro*^46^,^57^. Mammosphere formation and ALDH-positive cells were suppressed with the same concentration of the drug, emphasizing the ability of C1 to target breast CSCs. BCSC-hypothesis therapies involve either killing BCSCs or targeting the self-renewal signaling pathways of BCSCs^17^,^50^,^58^. IMD-0354 is a chemical agent that targets CSCs populations^59^. The chemical agent LDE225 was reported to inhibit prostate CSCs by targeting the hedgehog signaling pathway. Curcumin, a bioactive compound, inhibits CSCs in colonic and pancreatic cancer cells by targeting the Wnt and Notch self-renewal pathways of CSCs, respectively. β-catenin was downregulated in HeLa and HepG2 cells^21^. Based on our results, the Wnt/β-catenin self-renewal pathway was efficiently downregulated by complex C1 via GSK3β activation as a potential mechanism to target the MDA-MB-231 CSCs. The activation of cyclin D as an oncogene was reduced by its degradation through the phosphorylation by GSK-3β^21^. Therefore, decreasing the cyclin D1 expression level might be considered as a strategy in cancer therapy. Our results showed a reduction in cyclin D1 expression after treatment.

## Conclusion

Compound C1 induced cell death through apoptosis and G0/G1 arrest. The apoptotic induction of compound C1 was confirmed in this study by our results, including lactate dehydrogenase leakage, reactive oxygen species formation, translocation of phosphatidyl serine to the outer surface of the plasma membrane, mitochondrial membrane potential reduction, increased cytochrome *c* release, upregulation of Bax, downregulation of Bcl2 and HSP70, and the activation of initiator caspases. Moreover, C1 had the effect of targeting derived MDA-MB-231 CSCs, decreasing the number of mammospheres and ALDH-positive cells. Compound C1 also suppressed the Wnt/β-catenin self-renewal pathway through the downregulation of β-catenin, cyclin D1, and P-GSK3β and the upregulation of p-β-catenin (Ser33/Ser37/Thr41). In summary, this study supported the chemo-preventive features of complex C1 for breast cancer.

## Methodology

### Synthesis of benzyltin complex C1

**Figure 9:**
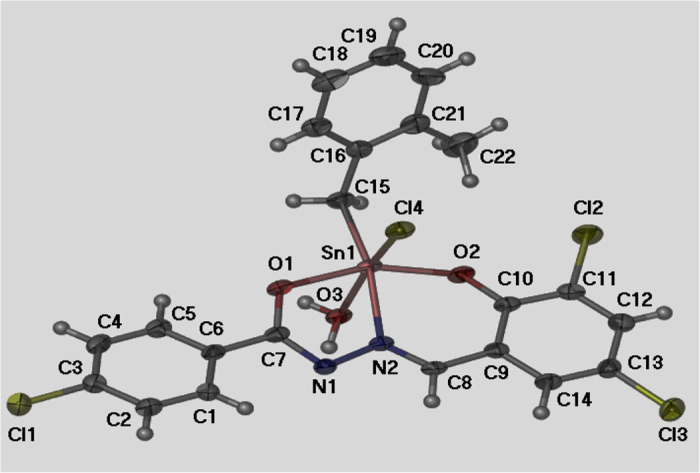
Molecular structure of compound C1.

The synthesis and characterization of [*N*-(3,5-dichloro-2-oxidobenzylidene)-4-chlorobenzyhydrazidato](*o*-methylbenzyl)-aquatin(IV) chloride, C1 (Fig. 9) was discussed in detail in previously published article^28^. This Schiff base compound was supplied by Dr. Lo Kong Mun from the Department of Chemistry at the University of Malaya in Kuala Lumpur, Malaysia.

### *In vivo* experiments and acute toxicity experiments

The University of Malaya Institutional Ethics Committee (Ethics no. 2015–180505/BMS/R/MAAH 2015/147) approved this experiment. Twelve female SD rats (150–180 g) were attained from the Experimental Animal House Facility from the Faculty of Medicine at the University of Malaya. We followed current procedures for the care of laboratory animals recommended by the National Academy of Sciences and published by the National Institutes of Health. All methods were performed in accordance with the relevant guidelines and regulations. The rats were divided into four groups and were placed in cages that were labeled as: A (25 mg/kg of compound), B (50 mg/kg of compound), C (100 mg/kg of compound), and D (vehicle control group). The rats were fasted overnight (with unlimited access to water) prior to dosing. After fasting, each group was administered its respective compound by oral gavage. Over the subsequent 3 to 4 hours, they were not given any food. The animals were closely observed for 3 to 4 hours after the administration of the compounds to detect any toxicological symptoms over the next 14 days. Before euthanizing the animals on the 15th day, histological, hematological, and serum biochemical factors were evaluated.

### Cell viability assay

The MDA-MB-231 (human breast adenocarcinoma cell line) cells used in this study were obtained from the American Type Cell Collection (ATCC, Manassas, VA, USA). Cells were cultured and maintained at the Roswell Park Memorial Institute in 1640 medium at 37 °C in a humid atmosphere with 5% CO_2_. The medium was supplemented with 10% fetal bovine serum (FBS) and 1% penicillin-streptomycin. To investigate the cytotoxicity of cisplatin, a positive control, to MDA-MB-231, cell viability assay was completed by MTT assay as previously described^28^. Briefly, cells were seeded onto 96-well culture plates at a density of 1 × 10^4^ cells/well and were treated with cisplatin at different concentrations. The cell viability was measured at absorbance of 570 nm using a microplate reader. The absorbance values were stated as percentages of controls, yielding percentage cell viability after 48 hours of treatment with cisplatin. The concentration of cisplatin with 50% cell growth inhibition was defined as the IC_50_ value.

### AO/PI Acridine Orange propidium iodide double staining

The morphological figures of apoptosis were studied using AO/PI dye. First, a concentration of 1 × 10^6^ of MDA-MB-231 cells was grown in 25 cm^2^ culture flasks. After 24 hours, they were treated with an IC_50_ concentration of compound C1 (2.5 μg/mL), based on a MTT assay discussed in a previous study^28^. After 48 hours of incubation in an atmosphere of 5% CO_2_ at 37 °C, both the treated and untreated cells were centrifuged at 1800 rpm for 5 minutes. The supernatant was discarded, and the pellet cells were rinsed using phosphate-buffered saline. Subsequently, 10 μg/mL of 1:1 AO/PI mixture, including 10 μg/mL acridine orange and 10 μg/mL propidium iodide, was used to suspend the pellet cells. Next, a fluorescence microscope (Zeiss Axioskop 2 plus, Germany) was used to analyze the morphological changes in cells. Green cells with intact nuclei indicated viable cells, while green cells with condensed nuclei indicated early apoptotic cells. Red fluorescence indicated the late apoptotic and necrotic stages, including either condensed or intact chromatin, respectively.

### LDH assay

The leakage of dehydrogenase (LDH) into the culture medium was measured to observe the growth-inhibiting effects of complex C1 against MDA-MD-231 cells. Treated cells with diverse concentrations of complex C1 and Triton X-100 (positive control) were incubated for 48 hours. Next, the medium was centrifuged at 3000 rpm for 5 minutes to obtain the supernatant. The supernatant was transferred to a new 96-well plate. One hundred microliters of the LDH reaction was added to each well and was incubated for 30 minutes at room temperature before measuring absorbance using a Tecan Infinite ^®^ 200 pro (Tecan, Männedorf, Switzerland) microplate reader at 490 nm. The volume of formazan salt generation and the intensity of the red color indicated the LDH activity in both the treated and untreated cells. The LDH release level in the treated cells was reported as a percentage of the positive control.

### DNA laddering assay

MDA-MD-231 cells at a concentration of 1 × 10^6^ were grown and treated with an IC_50_ concentration of compound C1 (2.5 μg/mL) based on the MTT assay published in a previous study for 24, 48, and 72 hours^28^. An equal number of untreated cells were treated as a negative control. The cells were centrifuged to collect the pellets (1000 rpm, 5 minutes). The pellets were used to isolate apoptotic DNA based on the protocol recommended by the Suicide Track^TM^ DNA Ladder Isolation Kit (Calbiochem, cat. no. AM41). Electrophoresis was used to analyze the extracted DNA on a 1.5% agarose gel. A mixture containing 21 μL of the samples and 5 μL of loading dye was loaded in the wells before running at a constant level of 50 V. The gel was stained with GelRed Nucleic Acid Gel Stain and was observed using a transilluminator.

### ROS assay

The intracellular ROS generation was evaluated using 2′,7′-dichlorofluorescein diacetate (DCFH-DA). Based on our procedure, the DCFH-DA dye was dissolved in 2.5 mL of DMSO to prepare 5 mM DCFH-DA stock solution. A 5 μM working solution was prepared by diluting the stock solution in PBS. The cells were plated in 96-well transparent culture plates at a density of 1 × 10^4^ cells per well and were treated with an IC_50_ concentration of complex C1 (2.5 μg/mL) for 24, 48, and 72 hours. The cells treated with 1 mM H_2_O_2_ were used as positive control, and untreated cells were used as a negative control. Next, 50 μL of DCFH-DA working solution were loaded in each well after 24 hours. The cells were incubated at 37 °C for 30 minutes before they were fixed with 16% formaldehyde for 10 to 15 minutes. Subsequently, the plate was washed 3 times with PBS before using a fluorescence microplate reader to measure the fluorescence intensity with excitation and emission wavelengths of 485 and 530 nm, respectively.

### Caspase activity

Luminescence-based assay kits, including Caspase-Glo 8, Caspase-Glo 9, and Caspase-Glo 3/7 (Promega), were used to measure the activity of caspases 3, 8, and 9. Cells at a density of 1 × 10^4^ were seeded on a 96 well Gernier transparent plate. After 24 hours, the cells were treated with an IC_50_ concentration of compound C1 (2.5 μg/mL), as discussed in the previous study^28^. A row with untreated cells and a row without cells were used as negative control and blank reaction, respectively. After 24 hours of treatment, 100 μL of caspase-Glo^®^ reagent, including caspase-Glo^®^ substrate, caspase-Glo^®^ buffer, and MG-132 inhibitor, were loaded in each well. A plate shaker was used to shake the plate at 300 to 500 rpm before it was incubated for 30 minutes at room temperature in the dark. Luminescence was measured using a microplate reader.

### Annexin V propidium iodide double staining assay

Further apoptotic effects of compound C1 against MDA-MB-231 cells was examined using an Annexin V FITC Apoptosis Detection Kit I (BD Pharmingen™, USA). One million cells were treated with an IC_50_ concentration (according to the MTT results from the previous study) of the compound (2.5 μg/mL) for 24, 48, and 72 hours^28^. Then, the cells were collected by centrifuging at 2000 rpm for 5 minutes. The supernatant was discarded, and the pellets were washed in 100 μL of binding buffer. Next, the cells were incubated for 15 minutes on ice in the dark with a mixture of 5 μL of PI and 5 μL of Annexin V. Then, 400 μL of binding buffer were loaded, and the analysis was completed using a BD FACSCantoTM flow cytometer (BD Bioscience, San Jose, CA, USA). Untreated cells were considered a negative control.

### Cell cycle assay

The effect of complex C1 on cell cycle distribution was examined using flow cytometry. In brief, cells at a density of 1 × 10^6^ were treated and collected for the Annexin V test. The pellets were fixed for one week by adding 500 μL of cold ethanol. Then, 1 mL of PBS-EDTA-BSA was added to the cells before centrifugation at 2000 rpm for 5 minutes. The PBS-EDTA-BSA solution was composed by adding 2 mg/mL EDTA to PBS and autoclaving, with 10 mL of this mixture used to liquefy BSA (0.1%) and to refine the BSA into PBS-EDTA. Subsequently, the washing buffer, composed of PBS (100 mL), BSA (1 gr), EDTA (20 mg), and sodium azide (100 mg), was used to wash the pellets immediately before adding a staining buffer that contained PBS (1 mL), PI (0.3 μg/mL), Rnase (50 μg/mL), Triton X-100 (1 μL/mL) and EDTA (0.37 mg/mL). A flow cytometer was used to analyze the cells after 30 minutes of incubation on ice.

### Cytotoxicity assay

The Cellomics Multiparameter Cytotoxicity 3 Kit was used to perform multiple cytotoxicity assays. According to their protocols, the cells were plated in a 96-well plate at a concentration of 10^6^ cells per plate and were incubated for 24 hours with a IC_50_ concentration of compound C1 (2.5 μg/mL) and cisplatin (and 0.9 μg/mL) for 24, 48, and 72 hours^28^. Next, the live cell staining solution (50 μL) was loaded in each well before incubation for 30 minutes at 37 °C. Then, 50 μL of paraformaldehyde 4% were added to each well to fix the cells, and PBS was used to wash the cells. Next, the cells were incubated with 50 μL of Primary Antibody Solution per well for 1 hour. The cells were further incubated for one hour with 50 μL of Secondary Antibody Solution at room temperature. Multiple features involved in cell health, such as the morphology of the nuclei, DNA content, mitochondrial membrane potential alterations, mitochondrial cytochrome *c* release, alteration in the permeability of cells, and cell loss, were simultaneously examined using an ArrayScan HCS system (Cellomics). The staining dyes in these experiments were Hoechst 33342 used as a nuclear dye, FITC used as a permeability dye, mitochondrial membrane potential dye (Cy5), and cytochrome *c* dye (Cy3). The cells in last row were considered a negative control. Before the experiment was conducted, all of the required solutions were composed according to standard procedures.

### Analysis of cytochrome *c* release

A Cytochrome *c* Releasing Apoptosis Assay Kit (Abcam, Cambridge, UK) was used to evaluate the release of cytochrome *c* from the mitochondria to cytosol after treatment with compound C1. The IC_50_ concentration of the compound (2.5 μg/mL) was used to treat MCF-7 cells at a density of 5 × 10^7^ cells for 24, 48 and 72 hours. Untreated cells were used as a control. Cells were collected by two cycles of centrifugation at 600 × *g* for 5 minutes at 4 °C and washing with 10 mL of ice-cold PBS. The pellet cells were re-suspended in 1.0 mL of 1X Cytosol Extraction Buffer Mix containing DTT and protease inhibitors before incubation on ice. Next, the cells were homogenized in an ice-cold Dounce tissue grinder. The homogenate was transferred to a 1.5 mL microcentrifuge tube and centrifuged at 700 × *g* for 10 minutes at 4 °C. The supernatant was transferred to a fresh 1.5-mL tube and was centrifuged at 10000 × *g* for 30 minutes at 4 °C. The supernatant was collected as the cytosolic fraction, which was then used to perform western blot analysis, as discussed in section on that analysis.

### Analysis of gene expression using real-time PCR

MDA-MB-231 cells at a concentration of 1 × 10^7^ was treated with an IC_50_ concentration of compound C1 (2.5 μg/mL) for periods of 24, 48, and 72 hours. The RNA extraction for both treated and untreated cells was performed using an RNeasy Plus Mini Kit (Qiagen, Germany). The 260/280 UV absorption proportions (Gen Quant 1300, UK) were used to measure RNA pureness and concentrations. The transcribed cDNA (1 μL) was prepared by converting the total RNA to cDNA using a Two-Step qRT-PCR Kit, High capacity RNA to CDNA- from Applied Biosystems (USA). The predesigned TaqMan primer and probe sets were obtained from Applied Biosystems to amplify reaction of the Bax, Bcl2, apoptotic marker genes, and ß-actin as a housekeeping gene. A Step One Plus Real Time PCR Machine was operated according this program: reverse transcriptase for 15 minutes at 48 °C, activation of ampli Taq gold DNA polymerase for 10 minutes at 95 °C, denaturation for 15 seconds at 95 °C and annealing for 1 minute at 60 °C. The denaturing and annealing phases were accomplished after 40 cycles. The Step One Plus Real Time PCR Machine and reagents, including the master mix and assays, were acquired from Applied Biosystems (USA). The comparative Ct (2^−ΔΔCt^) method was used to calculate the relative rates of PCR products according to the fold changes in each target gene, compared to the average of the ß-actin gene. The quantification of both target and reference genes was measured in the samples and referenced. Measurements were performed using GenEx software, and the RNA fold alterations were calculated by Data Assist software, version 3, from Applied Biosystems (USA).

### Isolation of Candidate Breast Cancer Stem Cells

A catcher tube-based cell sorter and a flow cytometer (FACSCalibur™, BD Biosciences, Franklin Lakes, NJ, USA) were used to isolate candidate MDA-MB-231 breast CSCs from MDA-MB-231 cells by sorting the CD44+/CD24-/low cell population. The cells at a concentration of 1 × 10^7^ cells/mL were stained using 20 μL of both CD44 antibody and CD24 antibody in a 5 mL tube. The CD44 mouse anti-human monoclonal antibody [clone MEM-85], fluorescein isothiocyanate [FITC] conjugate, CD24 mouse anti-human monoclonal antibody [clone SN3], phycoerythrin conjugate, mouse immunoglobulin G2b (FITC), mouse immunoglobulin G1 (R-phycoerythrin), were purchased from BD Biosciences. The tubes had been incubated in the dark at room temperature for 45 minutes. CellQuest Pro software was used to recognize the CD44+/CD24-/low cell population thru quadrant analysis.

### Non-adherent mammosphere formation assay

Isolated CSCs from MDA-MB-231 cells at a density of 1 × 10^3^ cells/mL of culture medium were grown in a 6-well ultralow attachment plate (TPP, Fisher Scientific, Waltham, MA, USA^60^. Serum-free Dulbecco’s Modified Eagle’s Medium/F12 medium (Lonza), which was complemented with particular supplements, enabled the cells to grow and form spheres. The supplements included B27 (Invitrogen), 1% antibiotic-antimycotic, 5 μg/mL insulin, 1 μg/mL hydrocortisone, 4 μg/mL gentamicin, 20 ng/mL epidermal growth factor (Gibco), and 20 ng/mL basic fibroblast growth factor (Gibco). A further 1 mL of fresh medium plus supplements was added to each well every other day. Incubation of the primary culture MDA-MB-231 CSCs with different concentrations of complex C1 (0, 1, 2, and 4 μg/mL) was conducted under mammosphere-forming conditions. The second-passage and third-passage cultures were prepared using a subculture of cells from compound C1-treated primary mammospheres for each individual group in the absence of complex C1. The *in vitro* number and size of the formed mammospheres were compared with those in the control group after 5 to 7 days. An Eclipse TE2000-S microscope (Nikon, Tokyo, Japan) was used to acquire images with MetaMorph software, version 7.6.0.0.

### Aldefluor enzyme test

High expression of ALDH enzyme in cancer cells is an indicator of cancer stem cell populations^54^. To identify the presence of mammary stem/progenitor cells in MDA-MB-231 cells, an Aldefluor enzyme assay was performed. Single MDA-MB-231 CSCs from cell cultures were incubated for 45 minutes (37 °C, 5% CO_2_). The procedures were performed based on the guidelines provided by the manufacture of Aldefluor™ (StemCell Technologies, Herndon, VA, USA) using BODIPY-aminoacetaldehyde (1 μmol/L per 10^6^ cells) as an ADH substrate. A flow cytometer was used to analyze the enzyme activity.

### Analysis of Protein expression using western blot

MDA-MB-231 cells were incubated with IC_50_ concentrations of compound C1 (2.5 μg/mL) for 24, 48, and 72 hours. Protein was extracted according to Nacalai Tesque protein extraction guidelines. Protein extracts (40 μg protein) were loaded for sodium dodecyl sulfate polyacrylamide gel electrophoresis (SDS–PAGE) and then were transferred to a polyvinylidene difluoride membrane (Bio-Rad Laboratories, Hercules, CA, USA). The membrane was then blocked at room temperature for 40 minutes with 5% non-fat milk in TBS-Tween Buffer 7 comprising 0.12 M Tris-base, 1.5 M NaCl, and 0.1% Tween 20. Subsequently, the probed membrane with the appropriate primary antibody was maintained at 4 °C for 12 hours before washing with a mixture of Tris-buffered saline and Tween 20 (TBST) buffer. The primary antibodies for the detection of apoptotic markers, including β-actin (1:5000), Bcl2 (1:1000), Bax (1:1000), and heat-shock protein (HSP) 70 (1:1000), were purchased from Santa Cruz Biotechnology (Santa Cruz, CA, USA), and the cytochrome *c* antibody (1:200) was purchased from Abcam (Cambridge, UK). To detect the Wnt/β-catenin self-renewal pathway, the primary antibodies, including β-catenin, phospho β-catenin S33+S37 (1:500), cyclin D1 (1:5000), GSK3β (1:5000), and phospho GSK3β (1:500), were obtained from Abcam (Cambridge, UK). A 1:1000 dilution of goat anti-mouse or goat anti-rabbit secondary antibodies combined with alkaline phosphatase (Santa Cruz Biotechnology, CA, USA) was used to wash the membrane before incubation for 30 minutes at room temperature. After incubation, the membrane was washed with TBST and was exposed to BCIP/NBT solution (Santa Cruz Biotechnology) to observe the protein bands.

### Statistical analysis

The data are expressed as the mean ± standard deviation (SD) of three different tests. Statistical analysis was conducted by one-way ANOVA using the Prism statistical software package (GraphPad Software, USA). Statistical analysis was defined as significant if *P < 0.05.

## Additional Information

**How to cite this article**: Fani, S. *et al*. Anticancer activity of a monobenzyltin complex C1 against MDA-MB-231 cells through induction of Apoptosis and inhibition of breast cancer stem cells. *Sci. Rep.* **6**, 38992; doi: 10.1038/srep38992 (2016).

**Publisher's note:** Springer Nature remains neutral with regard to jurisdictional claims in published maps and institutional affiliations.

## Figures and Tables

**Table 1:** Effects of compound C1 on blood tests.

Group	HGB (g/dL)	HCT (%)	RBC (10^6^ cells/μL)	MCV (fL)	MCH (pg)	MCHC (g/dL)	RDW (%)	WBC (10^3^cells/μL)	Platelet (10^3^ cells/μL)
**Vehicle**	15.46 ± 0.12	44 ± 0.00	10.63 ± 0.16	62.31 ± 0.38	17.35± 0.21	34.26 ± 0.15	18.43 ± 0.35	7.53 ± 0.19	998.83 ± 25.43
**25 mg/kg**	15.27 ± 0.15	44 ± 0.00	10.57 ± 0.19	62.66 ± 0.34	17.82 ± 0.23	34.17 ± 0.24	19.33 ± 0.53	7.45 ± 0.21	983.39 ± 27.52
**50 mg/kg**	15.20 ± 0.18	43 ± 0.00	10.42 ± 0.22	62.75 ± 0.21	17.53 ± 0.13	34.15 ± 0.17	19.25 ± 0.25	7.38 ± 0.15	995.27 ± 22.32
**100 mg/kg**	15.25 ± 0.13	43 ± 0.00	10.82 ± 0.17	61.73 ± 0.41	18.51 ± 0.42	34.23 ± 0.29	20.57 ± 0.46	7.25 ± 0.14	1002.72± 26.31

The values are expressed as the mean ± standard deviation (SD). There were no significant differences between the groups. Statistical analysis is defined as significant if *P < 0.05.

**Table 2:** Effects of compound C1 on liver function tests.

Group	Total Protein (g/L)	Albumin (g/L)	Globulin (g/L)	TB (μmol/L)	AP (U/L)	ALT (U/L)	AST (U/L)	GGT (U/L)
**Vehicle**	60.45 ± 0.72	9.66 ± 0.28	51.17 ± 1.19	2.21 ± 0.15	154.36 ± 5.29	50.42 ± 1.34	173.56 ± 5.24	3.31 ± 0.23
**25 mg/kg**	58.63 ± 0.39	8.42 ± 0.43	50.53 ± 1.12	2.17 ± 0.28	153.45 ± 5.71	45.65 ± 1.74	175.59 ± 6.28	3.43 ± 0.14
**50 mg/kg**	59.27 ± 0.25	8.51 ± 0.33	50.49 ± 1.16	2.13 ± 0.35	154.27 ± 5.39	45.31 ± 1.19	175.38 ± 5.41	3.55 ± 0.21
**100 mg/kg**	59.54 ± 0.31	8.77 ± 0.21	50.38 ± 1.23	2.19 ± 0.18	155.69 ± 6.54	46.76 ± 1.48	176.25 ± 6.32	3.62 ± 0.31

The values are expressed as the mean ± standard deviation (SD). There were no significant differences between the groups. Statistical analysis is defined as significant if *P < 0.05.

**Table 3:** Effects of compound C1 on renal function tests.

Group	Sodium (mM/L)	Potassium (mM/L)	Chloride (mM/L)	CO_2_ (mM/L)	Anion (mM/L)	Urea (mM/L)	Creatinine (μM/L)
**Vehicle**	138.37 ± 0.23	4.32 ± 0.26	103.42 ± 0.27	26.65 ± 0.36	13.32 ± 0.32	4.41 ± 0.36	31.51 ± 1.29
**20 mg/kg**	139.53 ± 0.34	5.15 ± 0.23	104.29 ± 0.38	26.54 ± 0.43	13.64 ± 0.29	4.72 ± 0.31	29.37 ± 1.54
**50 mg/kg**	138.37 ± 0.41	5.18 ± 0.14	103.22 ± 0.19	27.41 ± 0.26	14.57 ± 0.20	4.39 ± 0.12	30.44 ± 1.32
**100 mg/kg**	140.76 ± 0.32	4.27 ± 0.31	103.35 ± 0.43	27.72 ± 0.52	14.38 ± 0.28	5.48 ± 0.24	30.29 ± 1.26

The values are expressed as the mean ± standard deviation (SD). There were no significant differences between the groups. Statistical analysis is defined as significant if *P < 0.05.

**Table 4:** IC_50_ of compound C1 and cisplatin against the MDA-MB-231 cell line.

Agent	IC_50_ ± SD (μg/mL)	
	24 hours	48 hours
Compound C1	3.5 ± 0.50	2.5 ± 0.50
Cisplatin	1.3 ± 0.61	0.9 ± 0.49
